# Early administration of nirmatrelvir/ritonavir leads to faster negative SARS-CoV-2 nasal swabs than monoclonal antibodies in COVID 19 patients at high-risk for severe disease

**DOI:** 10.1186/s12985-024-02333-x

**Published:** 2024-03-20

**Authors:** Marta Colaneri, Giovanni Scaglione, Federico Fassio, Lucia Galli, Alessia Lai, Annalisa Bergna, Arianna Gabrieli, Maciej Tarkowski, Carla Della Ventura, Valeria Colombo, Laura Cordier, Davide Bernasconi, Mario Corbellino, Gianfranco Dedivitiis, Silvia Borghetti, Debora Visigalli, Salvatore Sollima, Giacomo Casalini, Giuliano Rizzardini, Andrea Gori, Spinello Antinori, Agostino Riva, Monica Schiavini

**Affiliations:** 1 Department of Infectious Diseases, Unit II, L. Sacco Hospital, ASST Fatebenefratelli Sacco, Milan, Italy; 2 Department of Public Health, Experimental and Forensic Medicine, Unit of Biostatistics and Clinical Epidemiology, University of Pavia, Pavia, Italy; 3 Department of Biomedical and Clinical Sciences, University of Milan, Milan, Italy; 4 Department of Infectious Diseases, Unit I, L. Sacco Hospital, ASST Fatebenefratelli Sacco, Milan, Italy; 5 Institute of Infectious Diseases & Tropical Medicine, III Division, ASST Fatebenefratelli Sacco, Luigi Sacco Hospital, Milan, Italy; 6 Pharmacy Unit, ASST Fatebenefratelli Sacco, Luigi Sacco Hospital, Milan, Italy; 7 Centre for Multidisciplinary Research in Health Science (MACH), University of Milan, Milan, Italy

**Keywords:** COVID-19, Monoclonal antibodies, Time to negativization, Immunosuppression, Nirmatrelvir/Ritonavir

## Abstract

**Purpose:**

Besides the well-established efficacy in preventing severe COVID-19, the impact of early treatments, namely antivirals and monoclonal antibodies (mAbs), on the time length to negativization of SARS-CoV-2 nasal swabs is still unclear. The aim of this study was to compare the efficacy of different early treatments in reducing the SARS-CoV-2 viral shedding, identifying a single drug that might potentially lead to a more rapid negativization of SARS-CoV-2 nasal swab.

**Methods:**

This was a single-centre, retrospective, observational study conducted at Ospedale Luigi Sacco in Milan. Data of high-risk COVID-19 patients who received early treatments between 23 December 2021 and March 2023 were extracted. The comparison across treatments was conducted using the Kruskall-Wallis test for continuous variables. Dunn’s test with Bonferroni adjustment was performed for post-hoc comparisons of days to negativization. Secondly, a negative binomial regression adjusted for age, sex, number of comorbidities, immunosuppression, and SARS-CoV-2 vaccination status was implemented.

**Results:**

Data from 428 patients receiving early treatments were collected. The majority were treated with Nirmatrelvir/Ritonavir and were affected by SARS-CoV-2 Omicron infection with BA.2 sublineage. The median length time to SARS-CoV-2 nasal swab negativization was 9 days [IQR 7–13 days]. We found that Nirmatrelvir/Ritonavir determined a significant decrease of the length time to SARS-CoV-2 nasal swab negativization compared to mAbs (p = 0.003), but not compared to Remdesivir (p = 0.147) and Molnupiravir (p = 0.156).

**Conclusion:**

Our findings highlight the importance of promptly treating high-risk COVID-19 patients with Nirmatrelvir/Ritonavir, as it also contributes to achieving a faster time to negative SARS-CoV-2 nasal swabs.

## Background

COVID-19 challenges currently lie in management of fragile patients, such as the elderly and those with underlying diseases who are more likely to experience a severe progression [1].

To improve the prognosis of such patients, it is recommended that timely antiviral and monoclonal antibodies (mAbs) treatments should be started as soon as possible [2].

While the individual clinical efficacy of these treatments has been widely confirmed [3], it is still unclear their possible contribution in reducing the time length to negativization of nasal swabs for SARS-CoV-2.

Furthermore, comparative studies between different mAbs and antivirals on this matter are lacking.

In these high-risk patients who are offered early treatments, the viral shedding might be long-lasting, both preventing the access to outpatients’ care services and leading to the emergence of new potentially resistant viral variants [4].

We aim to assess which early treatment had the greatest impact on time length to negativization among different mAbs and antiviral drugs in COVID-19 patients at high risk of developing a severe disease.

## Methods

### Study design

This was a single-centre, retrospective, observational study of outpatients with a confirmed diagnosis of COVID-19, referred to Ospedale Luigi Sacco, in Milan from December 2021 until March 2023.

Medical records of adult patients diagnosed with COVID-19 through a SARS-CoV-2 positive RT-PCR or a rapid antigen test from nasal swabs and consequently evaluated for administration of early treatments, were pseudo-anonymized and abstracted on standardized data collection forms.

All participants signed informed written consent, and the study was approved by the Ospedale Luigi Sacco Institutional Review Board (n.prot 2020/ST/049).

### Study participants

We extracted the demographic, virological, and clinical data of all COVID-19 patients eligible for early treatments according to the Italian Medicines Agency recommendations [5].

Specifically, these patients had paucisymptomatic COVID-19, namely not-hospitalized and without any oxygen requirement. Patients were included if they had both mild COVID-19 (symptoms of SARS-CoV-2 infection but without pneumonia and without requiring oxygen-therapy) and fitted in the AIFA (Agenzia Italiana del Farmaco) definition of being at high-risk for progression into the severe disease. Specifically, they must have at least one of the following conditions: age over 65 years old, presence of solid or haematological cancer, chronic kidney disease, chronic liver disease, chronic lung disease, uncontrolled diabetes, neurological disease, cardiovascular disease, mental health condition, obesity, cerebrovascular disease and being immunocompromised (AIDS, solid organ or blood stem cell transplantation, and all those conditions requiring the use of corticosteroids or other immunosuppressive medications). [https://www.aifa.gov.it/emergenza-covid-19].

Patients were followed up for 28 days after the end of the drug administration to determine whether any side effects had occurred and whether they had been hospitalized for COVID-19.

### Patients’ characteristics

#### The demographic data included gender and age

Virological data consisted of the date of the first positive and first negative diagnostic test for COVID-19, and the genotyping of the SARS-CoV-2 isolates.

Clinical data comprised the onset symptoms, the vaccination status, and the underlying comorbidities.

Treatment data concerned which antiviral (Remdesivir, Nirmatrelvir/Ritonavir, Molnupiravir) or mABs treatment (Sotrovimab, Casirivimab/Imdevimab, Tixagevimab/Cilgavimab, Bamlanivimab/Etesevimab) was administered and any potentially related adverse event.

Treatments were administered according to the AIFA guidelines available at that time, and the decision to prescribe antivirals or monoclonal antibodies was a nuanced process, considering factors such as oral vs. intravenous administration, potential drug interactions, treatment efficacy, and patient convenience. We believe this approach allowed for tailored and patient-centered care based on the evolving circumstances of each case. [https://www.aifa.gov.it/emergenza-covid-19]

### Outcome

The primary outcome of this study was to evaluate the impact of mAbs and antiviral drugs on the length of SARS-CoV-2 viral shedding, identifying a potential drug which might more rapidly lead to a SARS-CoV-2 negative nasal swab.

### Statistics

Continuous variables were described as median and 25th and 75th percentile, while categorical variables as frequency and percentage.

The time of negativization of the SARS-CoV-2 nasal swab was calculated in days, as the difference between the first positive swab, executed at the onset of symptoms, and the first negative swab.

Correlation between days of negativization and continuous variables were investigated through Spearman’s correlation test, while differences between dichotomous categorical variables through Mann-Whitney U test.

The comparison of variables across treatments was conducted using the Kruskall-Wallis test and Chi-squared test with Yates’ continuity correction. Dunn’s test with Bonferroni adjustment was performed for post-hoc comparisons.

All mAbs were taken together in account as “mAbs group”, while antiviral drugs were individually considered.

A negative binomial regression adjusted for age, sex, total number of comorbidities, immunosuppression, and SARS-CoV-2 vaccination status was implemented.

Statistical significance threshold was set at 0.05.

Analyses were performed in R-Studio software (v. 4.2.3).

## Results

Overall, we considered the data of 428 high-risk patients treated with early treatments in our outpatient clinic.

Their demographic, virological, and clinical characteristics are reported in Table 1.

**Table 1:** General characteristics of the patients

	Molnupiravir (*N*=69)	mAbs (*N*=135)	Nirmatrelvir/Ritonavir (*N*=166)	Remdesivir (*N*=58)	p-value
**Days of negativization**					
median [Q1, Q3]	9.00 [7.00, 12.0]	11.0 [7.00, 15.0]	8.00 [7.00, 12.0]	9.00 [7.00, 12.8]	0.014
min-max	4.00-26.0	4.00-44.0	4.00-42.0	4.00-38.0	
**Age**					
median [Q1, Q3]	79.0 [61.0, 84.0]	61.0 [50.5, 73.0]	64.0 [53.3, 77.0]	65.5 [55.0, 73.8]	<0.001
min-max	40.0-90.0	24.0-94.0	23.0-95.0	26.0-88.0	
**Sex**					
0	30 (43.5%)	62 (45.9%)	83 (50.0%)	26 (44.8%)	0.773
1Male sex 111	39 (56.5%)	73 (54.1%)	83 (50.0%)	32 (55.2%)	
**Anti-SARS-CoV-2 vaccination**					
No	4 (5.8%)	23 (17.0%)	9 (5.4%)	2 (3.4%)	<0.001
Yes	65 (94.2%)	112 (83.0%)	157 (94.6%)	56 (96.6%)	
**Number of vaccination doses**					
median [Q1, Q3]	3.00 [3.00, 3.00]	2.00 [2.00, 3.00]	3.00 [3.00, 3.00]	3.00 [3.00, 3.00]	<0.001
min-max	0-5.00	0-4.00	0-4.00	0-4.00	
**Virus variant**					
BA1	7 (10.1%)	44 (32.6%)	7 (4.2%)	15 (25.9%)	<0.001
BA2, BN, XBB, OM4	13 (18.8%)	23 (17.0%)	55 (33.1%)	11 (19.0%)	
BA3, BA4, BA5, BE, BQ, BF	22 (31.9%)	1 (0.7%)	45 (27.1%)	6 (10.3%)	
Delta	3 (4.3%)	34 (25.2%)	1 (0.6%)	2 (3.4%)	
NA	24 (34.8%)	33 (24.4%)	58 (34.9%)	24 (41.4%)	
**Chronic kidney disease**					
No	57 (82.6%)	120 (88.9%)	155 (93.4%)	52 (89.7%)	0.099
Yes	12 (17.4%)	15 (11.1%)	11 (6.6%)	6 (10.3%)	
**Cardiovascular disease**					
No	13 (18.8%)	73 (54.1%)	90 (54.2%)	22 (37.9%)	<0.001
Yes	56 (81.2%)	62 (45.9%)	76 (45.8%)	36 (62.1%)	
**Oncological disease**					
No	59 (85.5%)	111 (82.2%)	138 (83.1%)	48 (82.8%)	0.948
Yes	10 (14.5%)	24 (17.8%)	28 (16.9%)	10 (17.2%)	
**COPD**					
No	54 (78.3%)	115 (85.2%)	129 (77.7%)	41 (70.7%)	0.125
Yes	15 (21.7%)	20 (14.8%)	37 (22.3%)	17 (29.3%)	
Immunosuppression*					
No	61 (88.4%)	79 (58.5%)	95 (57.2%)	41 (70.7%)	<0.001
Yes	8 (11.6%)	56 (41.5%)	71 (42.8%)	17 (29.3%)	
**Obesity**					
No	59 (85.5%)	108 (80.0%)	137 (82.5%)	40 (69.0%)	0.092
Yes	10 (14.5%)	27 (20.0%)	29 (17.5%)	18 (31.0%)	
**Diabetes**					
No	50 (72.5%)	116 (85.9%)	144 (86.7%)	42 (72.4%)	0.008
Yes	19 (27.5%)	19 (14.1%)	22 (13.3%)	16 (27.6%)	
**Hepatic disease**					
No	68 (98.6%)	131 (97.0%)	157 (94.6%)	54 (93.1%)	0.325
Yes	1 (1.4%)	4 (3.0%)	9 (5.4%)	4 (6.9%)	
**Hemoglobinopathy**					
No	67 (97.1%)	133 (98.5%)	162 (97.6%)	58 (100%)	0.598
Yes	2 (2.9%)	2 (1.5%)	4 (2.4%)	0 (0%)	
**Neurological disease**					
No	65 (94.2%)	110 (81.5%)	146 (88.0%)	54 (93.1%)	0.029
Yes	4 (5.8%)	25 (18.5%)	20 (12.0%)	4 (6.9%)	
**Number of comorbidities**					
median [Q1, Q3]	2.00 [1.00, 3.00]	1.00 [1.00, 2.00]	1.00 [1.00, 2.00]	2.00 [1.00, 3.00]	0.003
min-max	0-4.00	0-5.00	0-4.00	0-4.00	

COPD chronic obstructive disease

* Immunodepression was defined as the presence of AIDS, solid organ or blood stem cell transplantation, and all those conditions requiring the use of corticosteroids or other immunosuppressive medications

Most of the patients were males (227, 53%), and the median age was 66 years [IQR 54.0, 77.0]. The most frequent risk factor for progression to severe COVID-19 was cardio/cerebrovascular disease (230, 53.7%), followed by immunosuppression (152, 35.6%).

Patients were mainly treated with antivirals (293, 68.4%), most frequently Nirmatrelvir/Ritonavir (166, 38.8%).

Among the other antivirals, Remdesivir was administered in 58 patients (13.5%) and Molnupiravir in 69 (16.0%). All patients completed the full treatment course, which was well tolerated, with no adverse events and no discontinuations reported.

Notably, no one was hospitalized for COVID-19 over the 28 days of follow-up time.

The genotypical analysis was performed only for 289 patients (67.7%). Sublineage Omicron BA.2 was the most frequently identified (102, 24.0%), followed by BA.1 (73, 17.2%). Differently, the Delta variant was the least prevalent (40, 9.3%).

The median length time to SARS-CoV-2 nasal swab negativization was 9 days [IQR 7–13 days].

The Kruskal-Wallis analysis showed that treatments had significantly different median length time to SARS-CoV-2 swab negativization (*p* = 0.016), and the Bonferroni adjustment for post-hoc comparisons further demonstrated that Nirmatrelvir/Ritonavir led to a significantly reduced median length time to SARS-CoV-2 nasal swab negativization compared to mAbs (*p* = 0.001).

Furthermore, the multivariable negative binomial regression analyses showed that treatment with Nirmatrelvir/Ritonavir exhibited a significant decrease in the length time to SARS-CoV-2 nasal swab negativization compared to mAbs (CI 0.08–0.29, *p* = 0.003), but not to Remdesivir (CI [-0.04–0.25] *p* = 0.147) and Molnupiravir (CI [-0.04–0.24] *p* = 0.156). Immunosuppression was significantly associated with a longer time to achieve the first negative SARS-CoV-2 swab (CI [0.08–0.30] *p* = 0.001), while no impact of sex (CI [-0.09- -0.30] *p* = 0.922), age (CI [-0.09-0.10] *p* = 0.436), number of comorbidities (CI [-0.01—0.08] *p* = 0.083) and vaccination status(CI [-0.26- -0.07] *p* = 0.257) was observed.

## Discussion

We showed that Nirmatrelvir/Ritonavir led to a faster time length to negativization of nasal swabs compared to mAbs when administrated to paucisymptomatic COVID-19 patients at high risk of progression to severe disease.

Despite the lack of studies comparing the efficacy of the different early treatments, Nirmatrelvir/Ritonavir is currently the first choice since it has more robust outcome data. In fact, Remdesivir requires a more complicated parenteral administration over 3 days, while the potential benefit of Molnupiravir is certainly more modest [6].

While the main goal is to prevent COVID-19 progression in high-risk patients with efficacious early treatments, the potential impact of these drugs in lowering viral shedding also warrants a more thorough comprehension. Although the factors that predispose to protracted SARS-CoV-2 positivity remain to be defined, several cases of long-lasting SARS-CoV-2 positive nasal swabs were reported especially in immunocompromised patients [7], for whom the presence of a long-lasting positive SARS-CoV-2 swab might extremely delay routine management, increase transmission, and lead to the emergence of resistant virus variants.

Therefore, choosing a drug that not only would avert a worse course of the disease but also acts on the time length to negativization of nasal swabs, might be valuable.

Our findings indicate that Nirmatrelvir/Ritonavir leads to a shorter time until the first negative test in high-risk COVID-19 patients, retaining its leading performance also in this critical task. This result was suggested by another sporadic evidence of lower median time to obtain a negative SARS-CoV-2 swab in patients treated with Nirmatrelvir/Ritonavir compared to Sotrovimab [8].

Differently, while a further study also implied a similar superiority in this target compared to Molnupiravir [9], ours failed to find a difference between antivirals.

The retrospective nature is the main limitation of this study; thus, the superiority of Nirmatrelvir/Ritonavir cannot be fully assessed by such means. Moreover, the lack of an established follow-up to assess the viral clearance also limits the validity of the findings. Finally, the patient populations undergoing distinct treatments were inherently non-comparable, primarily due to the selective prescription patterns.

Remdesivir were intentionally avoided in patients with severe kidney or hepatic failure. Nirmatrelvir/ritonavir was generally avoided in those patients concurrently using medications with potential interactions, as outlined in the latest available resources (https://www.covid19-druginteractions.org/checker). Regarding mAbs, since the surge of the Omicron variant, concerns arose regarding their effectiveness against all the circulating SARS-CoV-2 variants. Therefore, the decision to utilize mAbs was generally based on current and available virological data, introducing an additional layer of complexity to the treatment evaluation. Although the current policy required an initial follow-up at one week following the initial positive swab, many patients independently proceeded to do it at home, subsequently reporting the date of execution. Moreover, since viral variants were unfortunately available only for a subset of patients, their role in the timing of viral shedding could not be assessed.

We believe that our study provides valuable insights into this meaningful topic, enhancing the prominence of early treatment with Nirmatrelvir/Ritonavir, which is currently the most preferable drug, also in terms of obtaining a faster time length to negativization of nasal swabs.

**Fig. 1:**
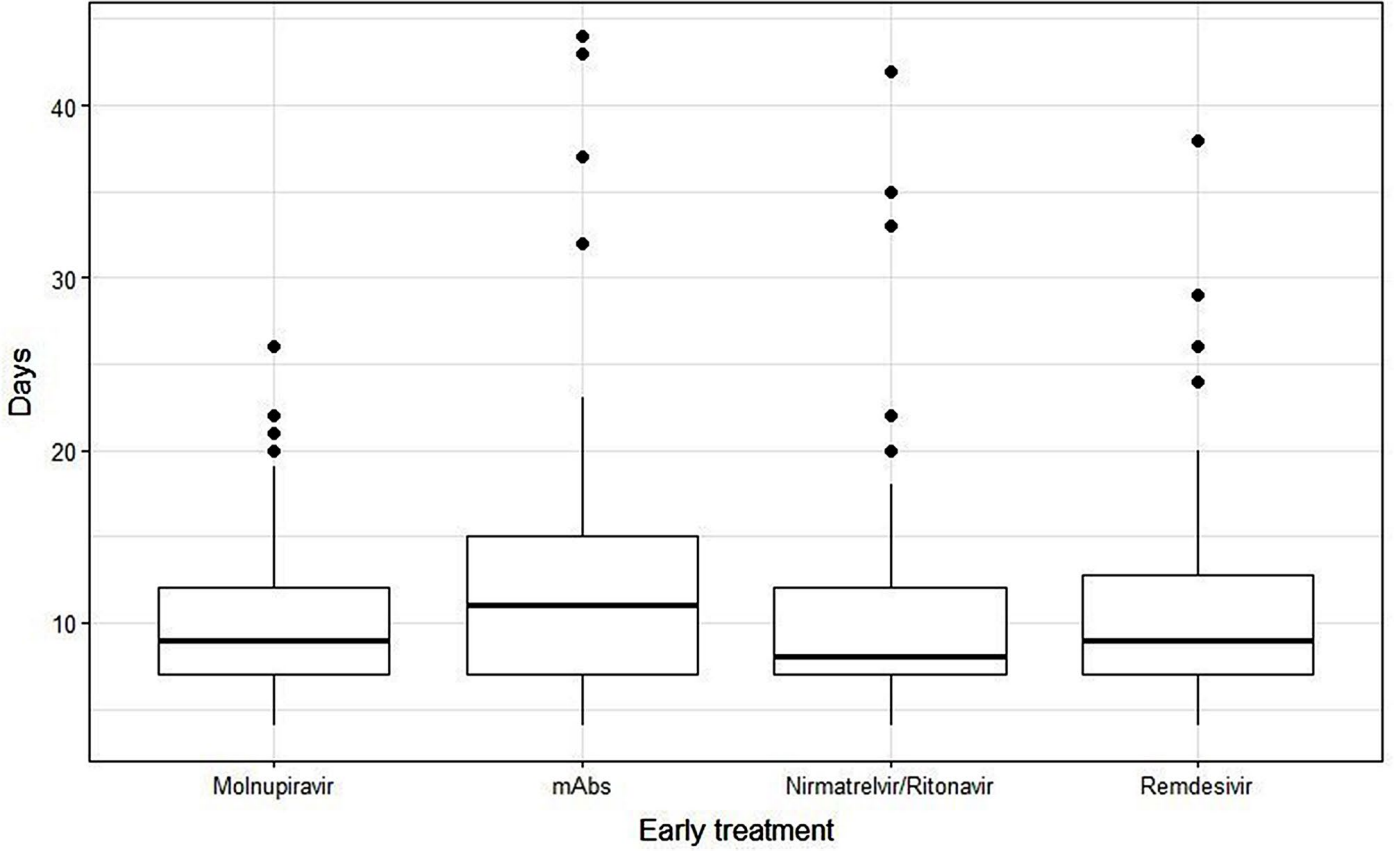
Impact of the different early treatments on the median length time to negativization of SARS-CoV-2 nasal swab

## Data Availability

All the materials are owned by the authors and/or no permissions are required. The dataset analysed during the current study is available from the corresponding author on reasonable request.
